# Physiological defensive modes to biologically induce drought tolerance in broccoli via inoculation with mycorrhiza and *Trichoderma*

**DOI:** 10.1186/s12870-025-06956-2

**Published:** 2025-07-19

**Authors:** Amr A. Metwally, Gamal S. Riad, Abdalla A. Ghoname, Sameh M. El-Sawy, Dina S. Salama, Layla Alkhawaga, Mostafa G. Shahin, Hani Saber Saudy, Nora A. AbdelMotlb

**Affiliations:** 1 Department of Horticulture, Faculty of Agriculture, Ain Shams University, Cairo, 11566 Egypt; 2 Vegetable Research Department, Agricultural and Biology Research Institute, National Research Centre, El Buhouth St, Cairo, 12622 Egypt; 3 Water Relations and Field Irrigation Department, Agricultural and Biological Research Institute, National Research Centre, El Buhouth St, Cairo, 12622 Egypt; 4 Botany Department, National Research Center, El Buhouth St, Cairo, 12622 Egypt; 5 Agronomy Department, Faculty of Agriculture, Ain Shams University, 11566, Cairo, Egypt

**Keywords:** Antioxidant defense, Broccoli curd yield, Cellular water balance, Osmo-regulators, Photosynthetic pigments

## Abstract

**Purpose:**

Recently, conserving irrigation water via application of deficit irrigation is a main priority, particularly in aridity and semi- aridity conditions. However, water deficiency is one of the major issues that hinder crop production worldwide. Also, knowledge regarding the physiological efficiency of microbial inoculation (MI) to shrink drought impacts in broccoli is unclear. We hypothesized that AMF and TRI fungi may have different mechanisms to modulate the physiological state and growth of broccoli to be more tolerant to drought stress.

**Methods:**

A field experiment in two seasons of 2023-24 and 2024-25 was conducted to assess the influence of MI on drought tolerance in broccoli. A mycorrhiza inoculum (AMF) and *Trichoderma* (TRI) inoculum were applied under 100, 80, 60 and 40% of irrigation requirements, IR (referred to as IR100, IR80, IR60 and IR40, respectively). Firstly, the MI was applied in the nursery after sowing the seeds in the seed trays, further, MI was applied two times, 28 and 38 days after transplanting (DAT) with the drip irrigation system, while the irrigation regimes started on 27 DAT. The treatments were designed in a strip-plot system in a complete randomized block with three replicates (trial unit size was 12 m^2^). At 70 DAT, soil plant analysis development (SPAD), membrane stability index (MSI), relative water content (RWC), proline content, enzymatic and non-enzymatic antioxidants and total antioxidant activity of broccoli leaves were assessed, while at 85 DAT the fresh and dry weight of shoot and root, leaf area and head yield were estimated.

**Results:**

Findings clarified that application of AMF and TRI inoculations under different levels of drought improved significantly (*p* < 0.05) SPAD, MSI and RWC compared to the corresponding control treatments, except AMF with IR60 for SPAD and TRI with IR60 for SPAD and MSI and with IR40 for SPAD. Under IR60 regime, AMF achieved the maximum improvements (*p* < 0.05) in peroxidase, polyphenol oxidase and superoxide dismutase, significantly equaling (*p* > 0.05) TRI for peroxidase. AMF×IR60 was the effective interaction for achieving the maximal values of total phenolic, total flavonoids and total antioxidant activity in broccoli, equaling (*p* > 0.05) TRI×IR60 combination in total phenolic. Compared to the counterpart control (CK) treatments the increases in proline content due to AMF or TRI applications amounted to 26.4 and 30.0% with IR80, 25.0 and 15.6% with IR60 and 36.6 and 32.5% with IR40, respectively. It is worth to observe that IR80×AMF combination achieved shoot dry weight and head yield values similar (*p* > 0.05) to IR100×AMF combination.

**Conclusion:**

Briefly, it can be concluded that microbial inoculations, specifically AMF, can relieve the injuries of drought. Practically, broccoli growers can save irrigation water by 20% with inoculating plants by mycorrhiza to maintain crop productivity and quality under water deficiency circumstances.

## Introduction

Broccoli is a cool-season vegetable crop that belongs to the Brassicaceae family. It originated in the Mediterranean region, cultivated for many centuries. Its edible part is the curd (head) that is harvested before blooming. Broccoli is recognized for its culinary usage and nutritional value, including a high content of vitamins C and K, fibers, and phytochemical compounds mainly the glucosinolates; which have antioxidant properties with health benefits, i.e., cancer prevention and health improvement [1–3]. Broccoli thrives in well-drained soils with pH ranges from 6.0 to 6.5, it has shallow roots and requires constant moisture without any fluctuations for optimal growth, yield and quality, since its sensitivity to drought stress conditions [4, 5]. Nevertheless, broccoli faces challenges in production, especially those related to water management and scarcity [6]. In Egypt, broccoli showed the maximum head yield when plants received 3800 m^3^ per hectare [7]. While, deficit water stress generated by limited irrigation inhibits photosynthesis, cell division, cell elongation, respiration, and stomatal movement, which in turn influences crop productivity [5, 8–11].

Climate change generates adverse environmental conditions such as drought, salinity, heat, etc. representing abiotic stressful factors on crop plants [12–15]. This ultimately impacts the plant’s growth, development, and productivity and deterioration of the acreages of arable lands [16–19]. Drought, as a consequence of climate change, is a major constraint on plant cultivation and productivity worldwide, affecting the guaranty of food [20–23]. The reduction in crop yields caused by drought is expected to surpass the combined losses attributed to all other factors contributing to yield decline [24]. Drought arises from a decline in rainfall, water scarcity and increment at evaporation rate [25, 26]. The influences of drought stress are determined according to the duration of time, growth stage and genetic tolerance of plants. By 2050, drought stress is anticipated to cause significant plant growth issues on over 50% of arable land [27]. At plant cell level, drought stress accumulates reactive oxygen species (ROS) at a level that exceeds the ability of the ROS scavenging systems, leading to damage the cell membrane integrity as well as oxidative destruction of the cell structure, physiological and metabolic disorders [28–32]. The hazardous impacts of ROS rely on the degree of oxidation of cell constituents; lipids, proteins, carbohydrates, photosynthetic pigments, DNA and nucleic acids [33]. Drought conditions negatively reduce the activity of RuBisCO as well as the photosystem II efficiency, while disturbing the electron transport chain leading to a decrement in the photosynthesis rate [34, 35]. Drought stress hinders the synthesis of photo assimilates, and the main reason for the decline in their production is the reduction of chlorophyll pigment and the synthesis of pigment intermediates [36–38]. Moreover, drought reduces the uptake and utilization of essential nutrients i.e., magnesium, which is a crucial component of the chlorophyll pigment molecule [39–41], therefore, crop yield and quality decline [42, 43].

Plants try to utilize many techniques to cope with this stress, by evading or increasing the ability to tolerate it; plants try to enhance water absorption by modifying the root system and decreasing the transpiration rate [44]. Also, soil microbial communities are increasingly recognized for their role in improving plant resilience [45–47]. In response to various environmental stressors, plants actively alter their microbiomes to recruit beneficial microbes that help them adapt and survive under challenging conditions [48–50].

Several techniques and approaches can alleviate drought stress in plants, such as developing drought-tolerant genotypes, using exogenous antioxidants, i.e., glutathione, salicylic acid, ascorbic acid, etc. [51–55]. Nowadays, the utilization of microbial inoculation (MI); bacteria and fungi, is an innovative technique implemented in agriculture to enhance plants’ resistance to abiotic and biotic stresses [56–61]. This approach can play a significant role in developing systems of sustainable agriculture since it makes plants more resilient to environmental climate problems [62].

Among these microorganisms, mycorrhiza (AMF) and *Trichoderma* (TRI) fungal species are particularly noteworthy. AMF make a symbiosis with plant roots, hence it provides various benefits to crops, including enhancing nutrient and water absorption, stress tolerance, and plant growth [63]. AMF are ubiquitous that are found associated with around 72% of terrestrial plants [64].

The AMF hyphae increase the plant root surface area and the absorption zone, making it capable of reaching inaccessible soil water and nutrients [65–67]. AMF inoculations increase the stomatal conductance, CO_2_ influx, and photosynthesis process of the host plants [68–70]. Concerning the inoculation with AMF, the report of Poveda et al. [71] have stated that plants of Brassicaceae have lost their ability to form AMF symbiotic relationships during evolution, probably due to the loss of AMF symbiosis-related genes [72] and/or the plants’ ability to recognize AMF effectors that play a crucial role in establishing the symbiotic association [73]. However, other symbiotic fungal structures were formed and these plants considered to be rudimentary arbuscular mycorrhiza host (RAM) [74]. Also, arbuscules were observed to form in *Brassica oleracea* [75]. Moreover, the success of the AMF colonization to the Brassicaceae plant roots depends on the genus of the fungi, since the Gigaspora and Rhizophagus genus can form AM and RAM structures in non-host plants [76]. Also, Mohamed et al. [77] studied the influence of *Glomus* spp. inoculation on broccoli plants, and the mycorrhizal treatment was revealed to be the superior application in his study.

*Trichoderma* species are fungi found in the rhizosphere zone that can contribute to plant growth and development [78], and enhance systemic resistance in plants [79, 80]. Many investigations mentioned the role of these microorganisms in relieving the biotic and abiotic stresses via diminishing the damage caused by ROS by inducing the antioxidant enzymes activity [81, 82].

While AMF and *Trichoderma* spp. have been shown to mitigate drought stress in crops like wheat [83], tomato [84], and sugarcane [85], studies specifically addressing their combined or individual effects on broccoli under graded irrigation regimes remain scarce. The present work hypothesized that AMF and TRI fungi may have different mechanisms to modulate the physiological status and growth of broccoli to be more tolerant to drought stress. Therefore, the prospective impacts of AMF and TRI were examined on broccoli under diverse regimes of irrigation.

## Materials and methods

### Study site attributes

A field experiment was organized at the trial farm of the National Research Centre, Nubariya, Beheira Governorate, Egypt, during the 2023/2024 and 2024/2025 growing seasons. To determine physico-chemical profile of the soil, samples were gathered (30-cm soil depth) and analyzed [86, 87]. A saturated paste extract of the soil was prepared (1:1 soil to water ratio) in order to determine the soil pH using pH meter. Phosphorus content was measured spectrophotometrically using the ascorbic acid method. Potassium was measured by flame photometer. Sodium was measured by flame photometer. Chloride was measured by Mohr’s method. Calcium was measured by the Versenate (EDTA) method. The soil sample analysis is represented in Table 1.

**Table 1:** Physico-chemical profile of the trial soil in the experimental farm before broccoli cultivation

Property	Unit	Value
Mechanical analysis		
Coarse sand (%)	%	18.07
Fine sand (%)	%	75.70
Silt (%)	%	4.34
Clay (%)	%	1.89
Soil texture		Sand
Chemical analysis		
pH value		8.10
EC at 25^o^C	dS m^−1^	0.58
Calcium	meq L^−1^	1.47
Magnesium	meq L^−1^	0.45
Sodium	meq L^−1^	1.61
Potassium	meq L^−1^	0.21
Chloride	meq L^−1^	1.25
Carbonate	meq L^−1^	-
Bicarbonate	meq L^−1^	1.33
Sulfate	meq L^−1^	1.16

In both seasons, all cultural practices of cultivation (fertilization, weeding, and pest control) were performed as recommended by the Egyptian Ministry of Agriculture and land reclamation and were kept normal and uniform for all the treatments. The Fertilization rates of N, P and K was 270, 250 and 150 kg/ha, respectively.

### Trial procedures

This study investigated the effects of microbial inoculation (MI) with arbuscular mycorrhiza (AMF) and Trichoderma fungi in comparison with non-inoculation (control) on broccoli plants grown under different drought stress conditions, expressed in irrigation regimes. Four irrigation regimes were applied at 100%, 80%, 60%, and 40% of irrigation requirement (IR) which denoted as IR100, IR80, IR60 and IR40. The irrigation regimes started at 27 days after transplanting (DAT).

The treatments were designed in a strip-plot system in a complete randomized block with three replicates. Irrigation regimes occupied vertical plots, while the microbial inoculation was applied in the horizontal plots. The trial unit size was 12 m^2^ (4 m × 3 m), and the four irrigation regimes were separated by a 3 m boundary.

Based on the equations of FAO Penman-Monteith [88], Doorenbos and Pruitt [89] and Vermeirer and Jopling [90] that were used to calculate the reference evapotranspiration (ETo), crop evapotranspiration (ETc) and IR, respectively. The irrigation requirements for the broccoli crop were precisely determined using the CROPWAT program, a widely recognized tool developed by the Food and Agriculture Organization (FAO) for calculating crop water needs. Daily climatic data for the study area, essential for CROPWAT calculations, were sourced from the FAO CLIMWAT 2.0 program, based on the meteorological stations included in the program [91]. The El Tahrier meteorological station, identified as the closest station to the Nubariya experiment site based on geographical coordinates, was selected and provided the necessary climatic inputs.

**Fig. 1:**
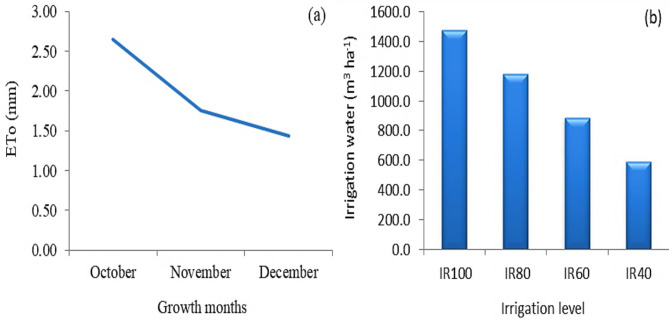
Changes in reference evapotranspiration (ETo) during growth months (**a**) and amounts of irrigation water under different irrigation levels (**b**) of broccoli grown at Nubariya, Beheira Governorate, Egypt (average of 2023/24 and 2024/25 seasons). IR80, IR60 and IR40: irrigation by 100, 80, 60 and 40% of irrigation requirements

Based on these calculations, daily water requirements and the depth of applied water were determined, and an irrigation schedule was established considering the broccoli crop type, planting date, and soil characteristics. To investigate the impact of drought stress, four distinct irrigation treatments were implemented, corresponding to percentages of the calculated water requirements. The required irrigation duration for each treatment (operating time of the irrigation system in minutes) and the intervals between irrigations were derived directly from the CROPWAT results. During broccoli growth months, ETo and water amount applied under different irrigation levels are illustrated in Fig. 1.

The irrigation system employed in this experiment consisted of four main experimental plots, each supplied with water from an irrigation tank. These tanks were filled up with water from a canal supplied from the Nile River. Irrigation for each plot was independently regulated by an electric solenoid valve, and the entire system was managed by an electronic control unit (Hunter, Hydrawise–Ready controller Pro-HC). Remote control of the system was facilitated by connecting the control unit to the Hydrawise mobile application, downloaded from the manufacturer’s website (Hydrawise mobile app).

The AMF inoculum involved a mixture of stock cultures of *Glomus* spp. and *Gigaspora* spp. isolate with 200 spore/ml. While, *Trichoderma* inoculum contained a mixture of *T. afroharzianum* and *T. longibrachiatum* at 30 × 10^7^ cfu/ml. Both inoculums provided from the Microbiological Resources Centre, Faculty of Agriculture, Ain Shams University. Firstly, the MI was applied in the nursery after sowing the seeds in the seed trays, the MI were applied by watering the trays with a suspension of the inoculant that was prepared by adding 1 L of the inoculant to 100 L of water for each of mycorrhizae and *Trichoderma*. Further, the MI was applied two times, 28 and 38 DAT in the field at the irrigation system at a rate of 7 L ha^−1^ of the inoculant.

Broccoli seeds (*Brassica oleracea* var. italica) cv. Larsson RZ F1 (25–735) were obtained from Rijk Zwaan Company, Egypt. At the nursery, cavity seeding trays were filled up with media that contains a mix of peat moss, vermiculite and perlite 1:1:1 volume by volume ratio. After sowing the seeds, the MI were applied by watering the trays with a suspension of the inoculant that was prepared by adding 1 L of the inoculant to 100 L of water for each of mycorrhizae and *Trichoderma*. The seeds were sown on 25th August in the nursery for obtaining seedlings (45 day-old) to be transplanted in the open field. After preparation of the open field, the transplants were sown (one seedling per hill) in rows, 45-cm distance and 90-cm apart, on the 10th of October of the 2023 and 2024 seasons. The plants were irrigated using a drip irrigation system.

### Sampling and data recording

#### Physiological attributes

At 70 DAT, the newly fully expanded developed leaf were collected from five randomly chosen plants per treatment assess membrane stability index, relative water content, proline content, enzymatic and non-enzymatic antioxidants and total antioxidant activity of broccoli leaves were assessed.

#### Leaf greenness

The greenness (SPAD) of the newly completely extended leaves of five plants was recorded using a chlorophyll meter (SPAD-502; Konica Minolta Sensing, Inc., Japan), the measurements were taken at four locations on each leaf; two on each side of the midrib, and then averaged. The SPAD readings were processed according to Xiong et al. [92].

#### Leaf water status

The membrane stability index (MSI) was calculated as mentioned by Premachandra et al. [93], leaf discs (2 cm in diameter) from fully expanded leaves were and placed in 30 ml of distilled water and allowed to stand in the dark for 24 h at room temperature. The electrical conductivity of the bathing solution was determined at the end of the incubation period (EC1) using a digital EC meter (Spectrum Technologies Inc., USA). Vials were heated in a temperature-controlled water bath at 95 °C for 20 min and then cooled to room temperature and the electrical conductivity (EC2) was again measured. MSI was calculated using formula 1.

1 $$\:MSI\:\left(\%\right)=\left[1-\left(\frac{\text{E}\text{C}1}{\text{E}\text{C}2}\right)\right]\times\:100$$

While the RWC of the leaves was estimated as described by Weatherley [94], 10 leaf discs (2 cm in diameter) obtained from young, fully expanded leaves on the third leaf from the plant apex on different plants were utilized. Leaf discs were weighed in order to determine the fresh weight (FW), then put in distilled water inside a covered Petri plate for 24 h. To determine the turgid weight leaf (TW), and the samples were then oven dried (72 h) to record their dry weight (DW). Values were used to calculate RWC using formula 2.

2 $$\mathrm{RWC}\;\left(\%\right)\;=\;\frac{\mathrm{FW}\;-\;\mathrm{DW}}{\mathrm{TW}-\mathrm{DW}}\times\;100$$

#### Enzymatic antioxidants

A fresh leaves sample of 0.2 g was grinded in 0.1 M ice-cold sodium phosphate (4 mL) buffer (pH 7.0) comprising 1% of polyvinylpyrrolidon and 0.1mM EDTA (w/v). Subsequently, at 4 °C, the mixture was centrifuged at 10.000× g for 20 min. The activities of peroxidase, POX [95], polyphenol oxidase, PPO [96], and superoxide dismutase, SOD [97] were measured.

#### Non-enzymatic antioxidants

The total phenolic (TP) content [98] and total flavonoid (TF) content [99] were assayed in leaves dry matter using a spectrophotometer. Further, the total antioxidant activity (DPPH) was determined based on the method illustrated by Brand-Williams et al. [100].

#### Proline content

Proline content was determined according to the method of mentioned by Habibi et al. [101, 102]. Fresh plant material (0.5 g) was homogenized in 10 mL of 3% (w/v) sulfosalicylic acid. The homogenate was centrifuged at 10,000 rpm for 10 min at room temperature, and 2 mL of the resulting supernatant was mixed with 2 mL of acid ninhydrin reagent and 2 mL of glacial acetic acid in a test tube. The mixture was incubated in a boiling water bath for 1 h and then immediately transferred to an ice bath to terminate the reaction. After cooling, 4 mL of toluene was added, and the mixture was vortexed for uniform mixing. The upper toluene layer was carefully separated and its absorbance was measured at 520 nm using a UV-Vis spectrophotometer. Proline concentration was quantified by comparison to a standard curve of L-proline.

#### Agronomic attributes

On the first of January (85 DAT) in both seasons, a random sample of five plants from the inner row of each experimental plot were collected to assess root fresh weight (RFW) and shoot fresh weight (SFW), and the average leaf area (cm^2^) that was calculated as relation between area unit and fresh weight of leaves [103] using formula 3.

3 $$\:\text{L}\text{e}\text{a}\text{f}\:\text{a}\text{r}\text{e}\text{a}\:=\:\frac{\text{D}\text{i}\text{s}\text{k}\:\text{a}\text{r}\text{e}\text{a}\:\times\:\:\text{d}\text{i}\text{s}\text{k}\:\text{n}\text{m}\text{b}\text{e}\text{r}\text{s}\:\times\:\:\text{l}\text{e}\text{a}\text{v}\text{e}\text{s}\:\text{f}\text{r}\text{e}\text{s}\text{h}\:\text{w}\text{e}\text{i}\text{g}\text{h}\text{t}}{\text{D}\text{i}\text{s}\text{k}\text{s}\:\text{f}\text{r}\text{e}\text{s}\text{h}\:\text{w}\text{e}\text{i}\text{g}\text{h}\text{t}}$$

After that, these fresh weights were dried in an oven at 70ºC till a constant weight to measure the dried biomass weights root (RDW) and shoot (SDW). Finally, the broccoli plants were harvested at the experimental plots then weighing the broccoli heads to estimate the average head weight, then the head yield ha^−1^ (HY) was estimated by multiplying the average head weight by the number of broccoli plants cultivated in the hectare.

### Statistical analysis

After performing Levene’s test to check the homogeneity of the data, the results demonstrated that the data’s normality and homogeneity are sufficient for running combined data analysis of the two seasons utilizing the mathematical model presented in formula 4 [104]. CoStat package program (Version 6.303; CoHort Software, USA) was utilized for carrying out the analysis of variance. At 5% level of probability, distinguishing the means of treatments were done relying on Duncan’s multiple range test. For unveiling the relation among the tested variables, heat map was depicted showing the coefficients of Pearson’s correlation.

4 $$\begin{aligned} \:\text{Y}ijk\:=&\:{\upmu\:}\:+\:{\uptau\:}i\:+\:{\upbeta\:}j\:+\:\left({\upbeta\:}{\uptau\:}\right)ij\:+\:{\upgamma\:}k\:+\:\left({\upbeta\:}{\upgamma\:}\right)jk\:\\&+\:\left({\uptau\:}{\upgamma\:}\right)ik\:+\:\left({\upbeta\:}{\uptau\:}{\upgamma\:}\right)ijk\:+\:{\upepsilon\:}ijk \end{aligned}$$

Where:

Y_*ijk*_ is response, µ is overall mean effect, *τ* is the treatment, *β* and *γ* are the blocks, and *β*_*j*_*∼* N(0, *σ*^2^_*β*_), *γ*_*k*_*∼* N(0, *σ*^2^_*γ*_), *ε*_*ijk*_*∼* N(0, *σ*^2^_*ε*_), all independent. {Assuming that the block factor to be random and the other factors to be fixed; independence between all errors}.

## Results

### Physiological attributes

#### SPAD, MSI and RWC

**Fig. 2:**
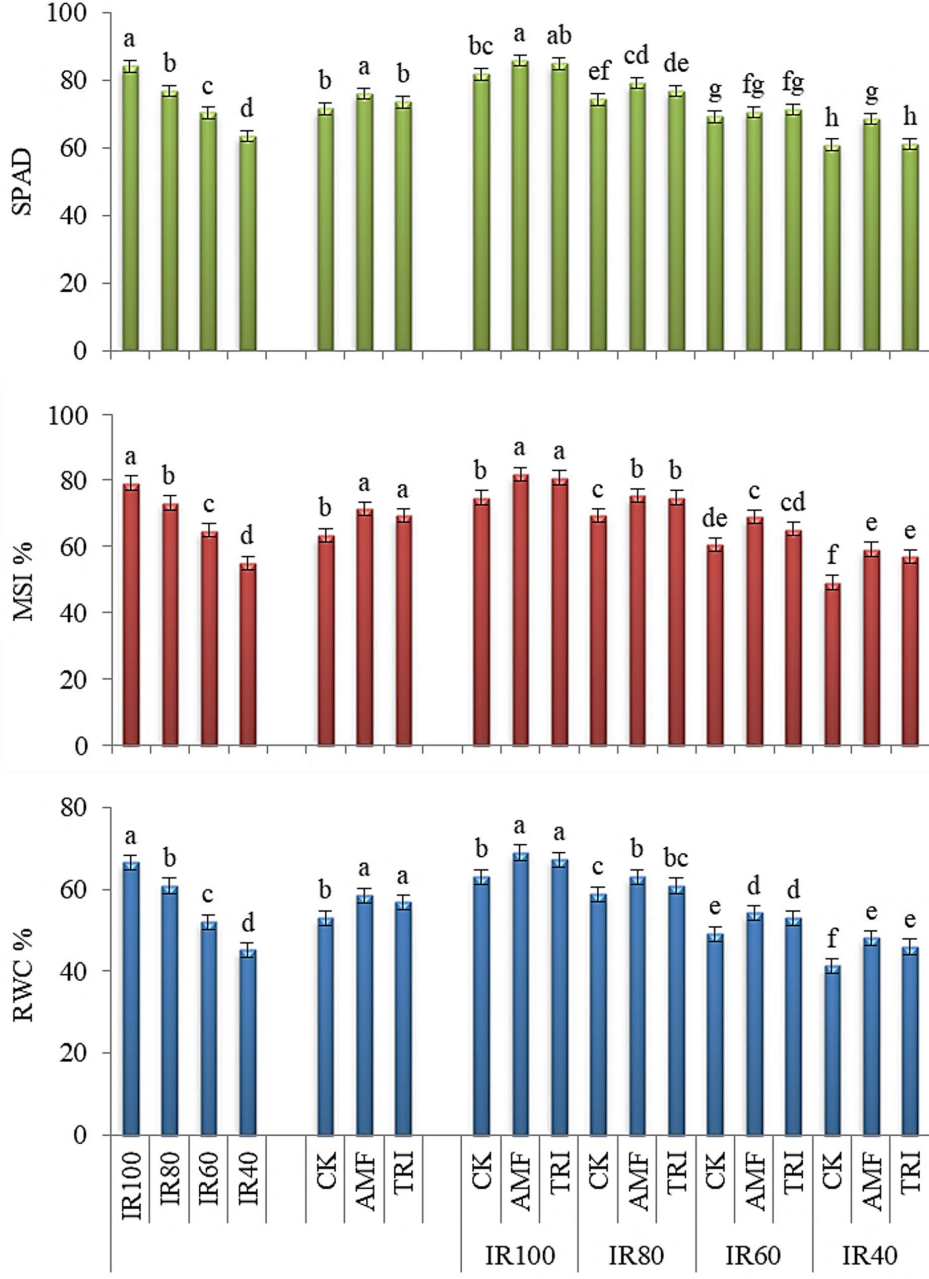
The change in leaf greenness (SPAD), membrane stability index (MSI) and relative water content (RWC) of broccoli with different irrigation requirements (IR) and microbial inoculations (MI). IR100, IR80, IR60 and IR40: irrigation by 100, 80, 60 and 40% of IR, respectively; CK, AMF and TRI: non-inoculation, inoculation by mycorrhiza and inoculation by *Trichoderma*, respectively

As shown in Fig. 2, supplying broccoli plants with IR100 recorded the highest significant (*p* < 0.05) values of SPAD, MSI and RWC, while significant decrements associated lower irrigation supply. Concerning the MI application, the highest significant (*p* < 0.05) values of the aforementioned attributes were obtained with the AMF application. TRI application showed also similar increases of for MSI and RWC as AMF application. The increases were 12.6 and 9.5% in MSI and 40.4 and 6.7% in RWC due to application of AMF and TRI, respectively, compared to the control treatment (CK).

Regarding the combination of IR and MI, the application of IR100 with either AMF or TRI showed the highest significant (*p* < 0.05) values of SPAD, MSI and RWC. IR100×AMF or TRI increased SPAD by 4.9 and 3.9%, MSI by 9.6 and 8.0% and RWC by 9.4 and 6.7%, compared to the corresponding control (IR100×CK), respectively. AMF and TRI under different levels of drought improved SPAD, MSI and RWC compared to the corresponding control treatments, except AMF with IR60 for SPAD and TRI with IR60 for SPAD and MSI and with IR40 for SPAD.

#### Enzymatic antioxidants

POX, PPO and SOD of broccoli significantly (*p* < 0.05) changed under different IR and MI treatments (Table 2). IR60 showed the highest values of POX, PPO and SOD, surpassing the other tested irrigation regimes. Both AMF and TRI outperformed the CK for increasing all enzymatic antioxidants activity, exhibiting 1.39 and 1.35 folds in POX, 1.34 and 1.18 folds PPO in and 1.80 and 1.61 folds in SOD, respectively. However, AMF was more influencer than TRI for stimulating the activity of PPO and SOD.

**Table 2:** The change in enzymatic antioxidants activity of broccoli with different irrigation requirements (IR) and microbial inoculations (MI)

		Peroxidase U/min g^−1^	Polyphenol oxidase U/min g^−1^	Superoxide dismutase U/min g^−1^
IR				
IR100		5.72 ± 0.2c	6.4 ± 0.2d	51.6 ± 3.8d
IR80		7.69 ± 0.3b	19.7 ± 0.6b	82.4 ± 4.4b
IR60		8.73 ± 0.4a	22.8 ± 0.9a	100.9 ± 5.2a
IR40		7.86 ± 0.4b	18.4 ± 0.5c	63.4 ± 4.7c
MI				
CK		6.00 ± 0.2b	14.3 ± 1.1c	50.6 ± 3.3c
AMF		8.36 ± 0.4a	19.3 ± 1.6a	91.5 ± 4.7a
TRI		8.14 ± 0.3a	16.9 ± 1.3b	81.7 ± 4.4b
IR×MI				
IR100	CK	4.99 ± 0.1 g	5.9 ± 0.2f	31.6 ± 0.9j
	AMF	5.54 ± 0.3 fg	6.8 ± 0.2f	67.6 ± 2.0 g
	TRI	6.61 ± 0.5de	6.6 ± 0.4f	55.6 ± 3.1 h
IR80	CK	6.38 ± 0.5d-f	16.5 ± 0.3e	57.7 ± 1.1 h
	AMF	9.00 ± 0.5b	22.2 ± 0.6b	97.9 ± 2.9c
	TRI	7.69 ± 0.1c	20.4 ± 0.5c	91.7 ± 2.0d
IR60	CK	6.98 ± 0.5 cd	18.7 ± 0.5d	72.3 ± 1.8f
	AMF	10.06 ± 0.3a	27.2 ± 0.5a	123.1 ± 1.8a
	TRI	9.16 ± 0.5ab	22.4 ± 0.6b	107.4 ± 0.6b
IR40	CK	5.64 ± 0.2e-g	16.3 ± 0.6e	40.8 ± 1.02i
	AMF	8.85 ± 0.3b	20.8 ± 0.4c	77.3 ± 5.9e
	TRI	9.09 ± 0.3ab	18.1 ± 0.5d	72.1 ± 6.1f

IR100, IR80, IR60 and IR40: irrigation by 100, 80, 60 and 40% of *IR *Respectively CK, AMF and TRI: non-inoculation, inoculation by mycorrhiza and inoculation by *Trichoderma*, respectively

Under IR60 regime, AMF achieved the maximum improvements in POX, PPO and SOD, significantly equaling (*p* > 0.05) TRI for POX. However, TRI was the efficient application for enhancing POX under IR40 regime.

#### Non-enzymatic antioxidants

Total phenolic, total flavonoids and total antioxidant activity in broccoli significantly (*p* < 0.05) responded to IR and MI (Table 3). In this respect, IR60 was the potent treatment for recoding the highest values, exceeding the other IR regimes. AMF surpassed CK and TRI for stimulating the production of all non-enzymatic antioxidants.

**Table 3:** The change in non-enzymatic antioxidants activity of broccoli with different irrigation requirements (IR) and microbial inoculations (MI)

		Total phenolic (mg g^−1^)	Total flavonoids (mg g^−1^)	DPPH (µM Trolox g^−1^)
IR				
IR100		14.4 ± 0.3d	0.093 ± 0.004c	11.9 ± 0.4d
IR80		20.5 ± 0.6b	0.127 ± 0.006b	37.2 ± 1.9c
IR60		24.5 ± 0.6a	0.177 ± 0.009a	63.4 ± 3.7a
IR40		19.5 ± 0.7c	0.125 ± 0.008b	40.3 ± 3.4b
MI				
CK		16.9 ± 0.6c	0.102 ± 0.006c	25.8 ± 2.58c
AMF		21.8 ± 0.9a	0.156 ± 0.009a	48.0 ± 4.97a
TRI		20.5 ± 0.8b	0.133 ± 0.007b	40.7 ± 4.15b
IR×MI				
IR100	CK	13.5 ± 0.7i	0.078 ± 0.003i	10.4 ± 0.6 h
	AMF	15.0 ± 0.2gh	0.109 ± 0.003f	13.1 ± 0.5 h
	TRI	14.6 ± 0.3hi	0.093 ± 0.004 h	12.1 ± 0.7 h
IR80	CK	17.1 ± 0.4f	0.094 ± 0.003gh	27.1 ± 1.3f
	AMF	22.8 ± 0.4b	0.148 ± 0.005c	45.4 ± 1.4d
	TRI	21.5 ± 0.5 cd	0.137 ± 0.007d	39.1 ± 1.2e
IR60	CK	20.7 ± 0.5de	0.138 ± 0.011d	43.7 ± 1.1d
	AMF	26.9 ± 0.4a	0.218 ± 0.003a	79.3 ± 1.1a
	TRI	25.9 ± 0.1a	0.175 ± 0.006b	67.1 ± 2.3b
IR40	CK	16.1 ± 0.8 fg	0.097 ± 0.011 g	22.0 ± 2.2 g
	AMF	22.3 ± 0.9bc	0.151 ± 0.012c	54.2 ± 1.3c
	TRI	20.17 ± 0.3e	0.127 ± 0.008e	44.7 ± 1.7d

IR100, IR80, IR60 and IR40: irrigation by 100, 80, 60 and 40% of IR, respectively; CK, AMF and TRI: non-inoculation, inoculation by mycorrhiza and inoculation by *Trichoderma*, respectively; DPPH: total antioxidant activity.

AMF×IR60 was the effective interaction for achieving the maximal values of total phenolic, total flavonoids and total antioxidant activity in broccoli, equaling TRI×IR60 combination in total phenolic.

#### Proline content

**Fig. 3:**
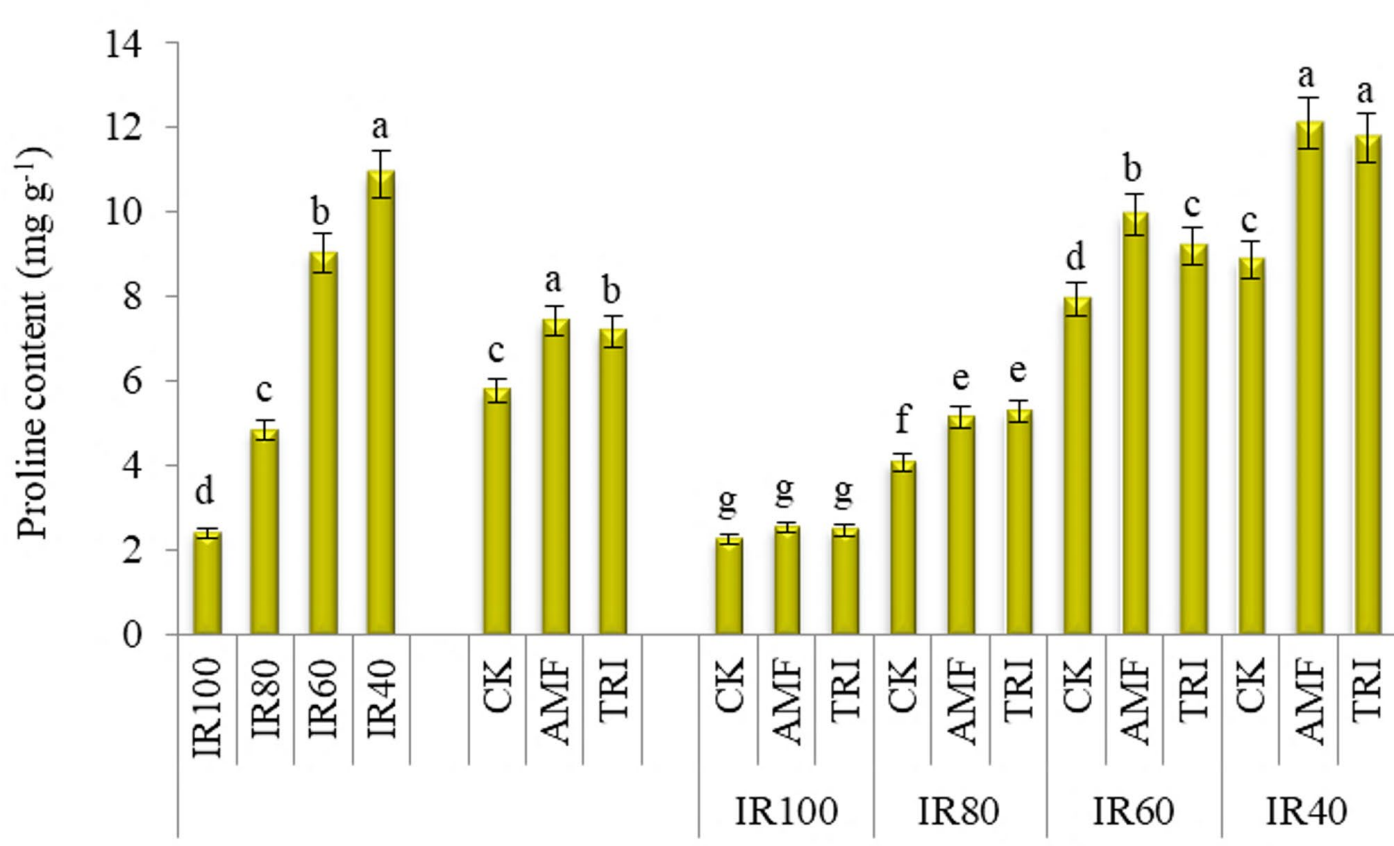
The change in proline content based on fresh weight of broccoli with different irrigation requirements (IR) and microbial inoculations (MI). IR100, IR80, IR60 and IR40: irrigation by 100, 80, 60 and 40% of IR, respectively; CK, AMF and TRI: non-inoculation, inoculation by mycorrhiza and inoculation by *Trichoderma*, respectively

As shown in Fig. 3, there was a progressive decrease in proline content with elevating soil moisture through increasing irrigation level. Thus, irrigation by IR40 and IR100 gave the lowest and highest proline content, respectively. Distinctive increases in proline content were obtained with AMF and TRI treatments outperforming (*p* < 0.05) the CK treatment by with higher superiority for AMF than TRI in this respect. Regarding the interactions, under different drought degree, AMF or TRI stimulated the production of proline, however the maximal values were recorded with AMF or TRI under IR40. Compared to the counterpart CK treatments the increases in proline content due to AMF or TRI applications amounted to 26.4 and 30.0% with IR80, 25.0 and 15.6% with IR60 and 36.6 and 32.5% with IR40, respectively. There was no noticeable (*p* > 0.05) change in proline content due to AMF or TRI applications under normal irrigation (IR).

#### Agronomic attributes

**Fig. 4:**
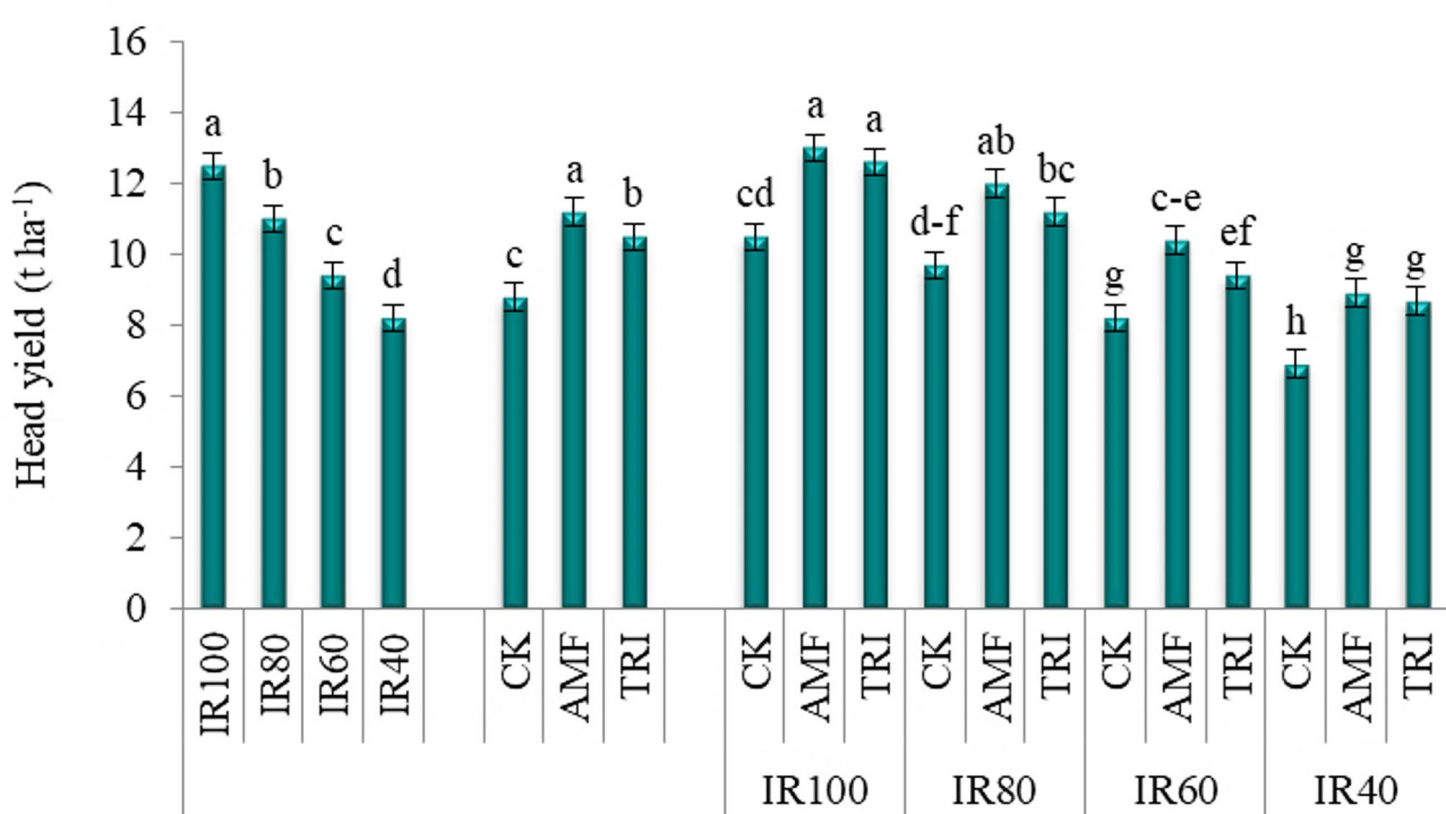
The change in head yield of broccoli with different irrigation requirements (IR) and microbial inoculations (MI). IR100, IR80, IR60 and IR40: irrigation by 100, 80, 60 and 40% of IR, respectively; CK, AMF and TRI: non-inoculation, inoculation by mycorrhiza and inoculation by *Trichoderma*, respectively

Analysis of variance proved that growth attributes (Table 4) and head yield (Fig. 4) of broccoli modified significantly as a result of varying irrigation amounts and microbial inoculation applications. In this context, all agronomic traits of broccoli declined with decreasing IR levels, with the lowest values ​​recorded at IR40. On the contrary, IR100 achieved the maximum increases in all agronomic traits, significantly equaling (*p* > 0.05) IR80 in root fresh weight and shoot dry weight. AMF application possessed the highest increases in all agronomic traits of broccoli outperforming the check treatment by 1.11, 1.25, 1.14, 1.32, 1.19 and 1.27 times in leaf area, root fresh weight, shoot fresh weight, root dry weight, shoot dry weight and head yield, respectively.

**Table 4:** The change in growth attributes of broccoli with different irrigation requirements (IR) and microbial inoculations (MI)

		Leaf area (cm^2^)	Fresh weight (g plant^−1^)		Dry weight (g plant^−1^)	
			Root	Shoot	Root	Shoot
IR						
IR100		1456.1 ± 34.0a	150.0 ± 4.1a	636.3 ± 16.3a	71.0 ± 2.8a	113.5 ± 3.1a
IR80		1356.8 ± 21.6b	141.6 ± 3.7ab	581.3 ± 13.9b	65.2 ± 2.2b	110.2 ± 2.8a
IR60		1280.9 ± 20.1b	135.4 ± 4.2b	530.0 ± 10.4c	61.8 ± 1.9b	104.0 ± 2.4b
IR40		1132.7 ± 22.3c	117.7 ± 3.4c	519.8 ± 8.6c	56.7 ± 2.0c	103.2 ± 2.3b
MI						
CK		1223.8 ± 32.2b	118.0 ± 3.0c	522.3 ± 11.5c	53.5 ± 1.1c	97.5 ± 1.5c
AMF		1364.7 ± 28.6a	148.4 ± 3.3a	599.6 ± 13.3a	70.7 ± 1.8a	116.5 ± 2.1a
TRI		1331.5 ± 29.8a	142.1 ± 3.0b	578.7 ± 13.6b	66.8 ± 1.9b	109.2 ± 2.0b
IR×MI						
IR100	CK	1371.8 ± 71.2c-e	129.9 ± 5.7d	572.3 ± 25.9d	58.0 ± 1.8 fg	100.3 ± 2.6d-f
	AMF	1512.8 ± 51.7a	164.3 ± 2.9a	682.9 ± 20.5a	80.1 ± 3.8a	126.9 ± 3.7a
	TRI	1483.7 ± 43.4ab	155.6 ± 2.2b	653.8 ± 17.6b	75.0 ± 3.2ab	113.4 ± 2.8bc
IR80	CK	1278.2 ± 34.7ef	125.5 ± 3.9d	529.5 ± 20.6f	55.5 ± 1.6 g	100.7 ± 3.4d-f
	AMF	1407.5 ± 30.6bc	151.8 ± 5.6bc	610.3 ± 16.5c	72.0 ± 2.4bc	117.5 ± 3.3ab
	TRI	1384.6 ± 26.8 cd	147.5 ± 3.8bc	604.1 ± 21.4c	68.1 ± 3.3 cd	112.3 ± 5.7bc
IR60	CK	1207.0 ± 27.1 fg	115.0 ± 3.9e	497.6 ± 15.5 g	52.4 ± 1.2gh	96.2 ± 2.9ef
	AMF	1335.5 ± 31.4c-e	147.9 ± 5.4bc	561.0 ± 12.4de	68.0 ± 1.4 cd	110.7 ± 3.5b-d
	TRI	1300.2 ± 25.4d-f	143.3 ± 3.4c	531.5 ± 17.5f	65.2 ± 2.8de	105.37 ± 4.3c-e
IR40	CK	1038.0 ± 15.4 h	101.4 ± 1.9f	489.7 ± 16.4 g	48.3 ± 1.9 h	93.0 ± 3.1 f
	AMF	1202.9 ± 17.1 fg	129.8 ± 4.2d	544.2 ± 9.1ef	62.8 ± 2.1d-f	110.9 ± 3.2b-d
	TRI	1157.4 ± 40.6 g	122.0 ± 3.4de	525.5 ± 9.9f	59.2 ± 3.1e-g	105.6 ± 1.6c-e

IR100, IR80, IR60 and IR40: irrigation by 100, 80, 60 and 40% of IR, respectively; CK, AMF and TRI: non-inoculation, inoculation by mycorrhiza and inoculation by *Trichoderma*, respectively.

Under well-water supply (IR100), both AMF and TRI showed similar remarkable enhancements in leaf area, root dry weight and head yield compared to the corresponding CK. However, AMF exhibited root fresh weight, shoot fresh weight and shoot dry weight values greater than CK and TRI. Under different deficit water (IR80, IR60 and IR40), AMF along TRI surpassed the counterpartying CK for improving all agronomic attributes. It is worth to observe that IR80×AMF combination achieved shoot dry weight and head yield values similar (*p* > 0.05) to IR100×AMF combination.

#### Correlation analysis

**Fig. 5:**
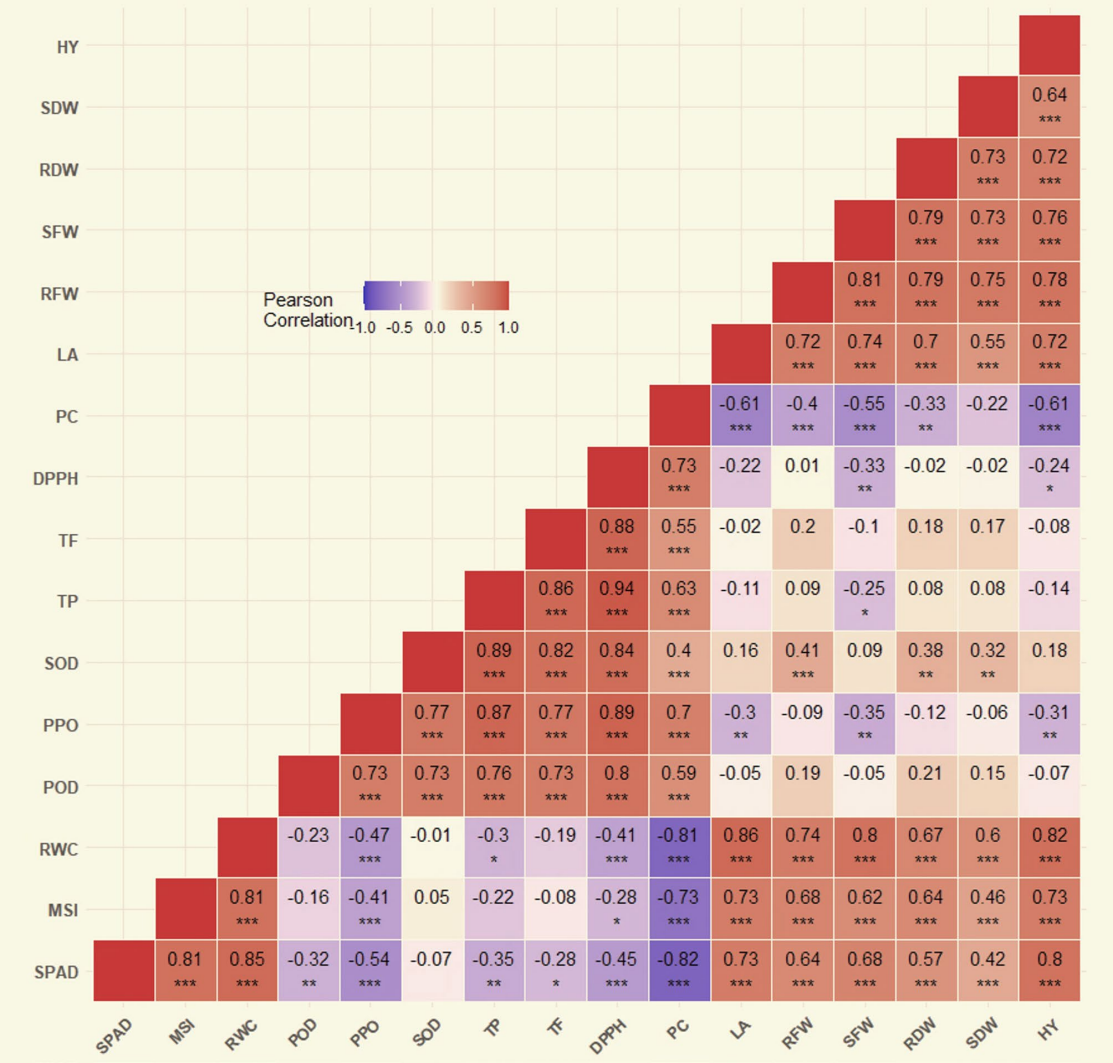
Heat map correlation coefficients between different pairs of broccoli characteristics estimated under different irrigation requirements and microbial inoculations. *, ** and ***: correlation is significant at 0.05, 0.01 and 0.001 level of significance. leaf greenness (SPAD), membrane stability index (MSI), relative water content (RWC), peroxidase (POD), polyphenol oxidase (PPO), superoxide dismutase (SOD), total phenolic (TP), total flavonoids (TF), total antioxidants (DPPH), proline content (PC), leaf area plant^−1^ (LA), root fresh weight plant^−1^ (RFW), shoot fresh weight plant-1 (SFW), root dry weight plant^−1^ (RDW), shoot dry weight plant^−1^ (SDW), head yield ha^−1^ (HY)

Correlation coefficients, expressed in heat maps, between each pairs of studied broccoli characteristics, i.e. SPAD, MSI, RWC, POD, PPO, SOD, TP, TF, DPPH, PC, LA, RFW, SFW, RDW, SDW, and HY were estimated (Fig. 5). The calculated correlation coefficients exhibited significant (*p* < 0.01) and positive associations between SOD and RDW, and SOD and SDW Moreover, there were positive and highly significant at *p* < 0.001 between SPAD and MSI, SPAD and RWC, SPAD and LA, SPAD and RFW, SPAD and SFW, SPAD and RDW, SPAD and SDW, SPAD and HY, MSI and RWC, MSI and LA, MSI and RFW, MSI and SFW, MSI and RDW, MSI and SDW, MSI and HY, RWC and LA, RWC and RFW, RWC and SFW, RWC and RDW, RWC and SDW, RWC and HY, POD and PPO, POD and SOD, POD and TP, POD and TF, POD and DPPH, POD and PC, PPO and SOD, PPO and TP, PPO and TF, PPO and DPPH, PPO and PC, SOD and TP, SOD and TF, SOD and DPPH, SOD and PC, TP and TF, TP and DPPH, TP and PC, TF and DPPH, TF and PC, DPPH and PC, LA and RFW, LA and SFW, LA and RDW, LA and SDW, LA and HY, RFW and SFW, RFW and RDW, RFW and SDW, RFW and HY, SFW and RDW, SFW and, SDW, SFW and HY, RDW and SDW, RDW and HY, SDW and HY.

On the contrary, the relations between SPAD and TF, MSI and DPPH, RWC and TP, TP and SFW, DPPH and HY significantly (*p* < 0.05) correlated with negative trends. However, the associations between SPAD and POD, SPAD and TP, PPO and LA, PPO and SFW, PPO and HY, DPPH and SFW, PC and RDW were negative and highly significant (*p* < 0.01). Further, there were negative and highly significant at *p* < 0.001 between SPAD and PPO, SPAD and DPPH, SPAD and PC, MSI and PPO, MSI and PC, RWC and PPO, RWC and DPPH, RWC and DPPH, RWC and PC, PC and LA, PC and RFW, PC and SFW, PC and HY.

## Discussion

At different levels of deficit water supply, broccoli plants exhibited sensitivity to drought as physiology and growth hindered due to irrigation by lower rates (IR80, IR60 and IR40) than normal (IR100). The sensitiveness to various drought levels was pronounced in degradation of plant pigments (SPAD), lowering MSI, RWC and changes in antioxidant defensive features. Thus, growth and yield declined owing to drought at different degrees. Remarkably, drought conditions negatively affect the vegetative growth, heads yield, quality of broccoli plants [4, 5]. Drought stress caused detrimental influences on plant growth, chlorophyll content, stomatal conductance, CO_2_ assimilation, nutrients absorption and acquisition and yield quality and production [5, 105]. Drought also disrupts the physiological and biochemical processes necessary for healthy growth by reducing the relative water content [1, 40, 106]. At plant cellular level, drought stress accumulates reactive oxygen species (ROS) at a level that exceeds the ability of the ROS scavenging systems, leading to damage the cell membrane integrity as well as oxidative destruction of the cell structure, physiological and metabolic disorders [29]. Further, the electron transport chain in mitochondria is impaired due to intemperate accumulation of ROS, disrupting respiration [34]. Relying on the degree of oxidation of cell constituents (lipids, proteins, carbohydrates, photosynthetic pigments, DNA and nucleic acids), the harmful effects was determined [33]. Accordingly, as the water deficit level increased, broccoli physiological status, growth, development and yield were adversely impacted. However, drought stress triggers the expression of genes related to stress responses and leads to the accumulation of specific metabolites that help in coping with the stress [107–110]. Therefore, progressive increases in enzymatic and and non-enzymatic antioxidants activity, in addition to proline content were observed with increasing drought level in broccoli.

On the other hand, the microbial inoculation application either AMF or TRI significantly enhanced SPAD, MSI, RWC, antioxidant modes, hence growth and yield attributes. The beneficial actions of AMF [83, 111] and TRI [84, 112] on plant growth behavior and productivity were documented. AMF hyphae increase root surface area and absorption zone, making the plant better able to access hard-to-reach soil water and nutrients such as phosphorus, nitrogen, and calcium [63]. Also, AMF enhances the stomatal conductance, in its turn, the CO_2_ assimilation increased at the inoculated host plants [70]. TRI had a promotional effect on plant growth, since the TRI-inoculated plants showed improving in stomatal conductance, relative water content and water use efficiency while reducing transpiration rate [113]. Further, TRI stimulate the production of some phytohormones at the inoculated plants; jasmonic acid and salicylic acid, that have roles at modulation of plant growth [114], Also, TRI can colonize the plant roots leading to the expansion of the root surface area [57]. In addition, the inoculation of TRI increases the root biomass and length by regulating the antioxidant enzyme activities, plant growth hormones, and the ascorbic acid–glutathione redox system [115].

Concerning role MI for inducing drought tolerance, AMF increases the antioxidant enzymes activity that alleviates the hazardous impacts of ROS that accumulate as a result of drought stress [63]. Further, AMF adjusts the hormonal balance at plant grown under drought stress conditions [116]. Also, AMF increase the N uptake and utilization and promoting amino acid accumulation in host plants [66, 117, 118]. Hence, AMF promotes the growth, development and productivity of broccoli plants grown under drought stress conditions. Regarding the beneficial mechanisms of TRI to mitigate drought, it has been found that *Trichoderma* spp. accumulates osmolytes such as proline, soluble sugar and polyamines content while increasing the antioxidant activity, protecting photosynthesis pigments and photosynthesis pathways from photo-oxidative damage [119, 120]. TRI can conserve the water content of the plant by inducing the stomatal closure through producing abscisic acid (ABA) [121]. In addition, TRI secretes various compounds and molecules; proteins, polysaccharides and antibiotics, some of these compounds act as plant growth regulators that induce the plant growth, also act as stimulants enhancing defense responses of plants, and increase the plant stress tolerance to abiotic stress [122]. *Trichoderma* species can mitigate drought stress in plants through various mechanisms, such as enhancing nutrient uptake, increasing chlorophyll pigment content, modifying antioxidant and proline metabolism, and improving photosynthesis process rate by promoting the stomatal conductance and gas exchange, water use efficiency and carboxylation efficiency [60, 85].

MI not only enhances the enzymatic antioxidants, but also the non-enzymatic antioxidants [57]. It has been found that the application of AMF and TRI increases the content of ascorbic acid, glutathione, α-tocopherol, carotenoids, and flavonoids [82, 83, 85]. In plant cells, glutathione (GSH) enhances the osmoregulation and water status. GSH is a substrate at glutathione peroxidase that plays a role in ROS detoxification [34]. GSH regulates gene expressions and transcription, and the signaling pathways by modifying the protein components via S-glutathionylation and/or S-nitrosoglutathione-mediated protein S-nitrosylation [123].

Accordingly, the enhancements resulting from MI applications in SPAD, MSI, RWC, growth, and yield of drought-stressed broccoli plants may be attributed to their role in stimulating the enzymatic antioxidant activity (POX, PPO and SOD), and non-enzymatic antioxidant activity (phenolic and flavonoids), in addition to proline accumulation as reported in the findings of this research.

Ultimately, this study highlighted on the physiological response of broccoli to *mycorrhizal* and *Trichoderma* fungi under irrigation regimes, involving drought stress. However, further investigations are needed to unveil potential modifications at the molecular level, contributing to deeper insights that could help vegetable breeders improve drought tolerance across different genotypes. Since drought may affect the colonization and efficiency of *mycorrhizal* and *Trichoderma* fungi, microbiologists are required to improve/discover new strains that are more adaptable to stress.

## Conclusions

The present work demonstrated that broccoli is a sensitive crop to deficit water at different levels. Thus, utilizing the eco-friendly approach is a significant practice to develop the physiological defense modes to enhance the ability to tolerate drought. The findings obviously showed that mycorrhiza and *Trichoderma* fungi are not only bio-source of nutrients but also possess diverse mechanisms to reliever the impacts of drought on broccoli. However, mycorrhiza was more efficient than *Trichoderma* in shrinking the detrimental influences of deficit water. Overall, the results revealed that mycorrhiza and *Trichoderma* fungi conferred drought tolerance to broccoli plants via synergistic mechanisms that include maintaining photosynthetic pigments, leaf water content, stabilizing the cell membranes, enhancing the activity of enzymatic and non-enzymatic antioxidants system, and elevating proline accumulation, thus improving yield under drought conditions. Although this study has physiologically disclosed the importance of mycorrhiza and *Trichoderma* fungi for mitigation of drought effects, still there are unclear molecular aspects need to be unveiled through future research. Since drought may affect the colonization and efficiency of *mycorrhizal* and *Trichoderma* fungi, microbiologists are required to improve/discover new strains that are more adaptable to stress.

## Data Availability

Data availability The datasets used and/or analyzed during the present investigation available from the corresponding author on reasonable request.
